# Management of Recurrent Weight Gain After Sleeve Gastrectomy: Comparative Effectiveness of Conversion Procedures Versus Obesity Management Medications

**DOI:** 10.1007/s11695-026-08727-w

**Published:** 2026-05-18

**Authors:** Tom Krauze, Avner Leshem, Yael Sofer, Andrei Keidar, Shai Meron Eldar, Adam Abu-Abeid

**Affiliations:** 1 Tel Aviv Sourasky Medical Center, Tel Aviv, Israel; 2 The Grays Faculty of Medical Health and Sciences, Tel Aviv University, Tel Aviv, Israel

**Keywords:** Weight gain, Sleeve Gastrectomy, Conversion surgery, Obesity Management Medications, Gastric Bypass, Long-term

## Abstract

**Background:**

Recurrent weight gain after sleeve gastrectomy (SG) is a common long-term challenge, often requiring additional intervention. Management options include conversion surgery—most commonly to Roux-en-Y gastric bypass (RYGB) or One-anastomosis gastric bypass (OAGB), or treatment with obesity management medications (OMMs). Comparative long-term data between these strategies remain limited. This study aimed to evaluate long-term outcomes of OMM therapy versus conversion surgery after SG for recurrent weight gain.

**Methods:**

A retrospective analysis of a prospectively maintained registry from a tertiary university hospital was conducted, including patients treated for recurrent weight gain after SG between 2014 and 2024. Patients underwent conversion surgery (OAGB or RYGB) or were treated with OMMs.

**Results:**

A total of 195 patients were included: OAGB (*n* = 69), RYGB (*n* = 62), and OMM (*n* = 64). Mean follow-up was 6.4 years. BMI at last follow-up differed significantly between groups (OMM 33.3 ± 5.1 kg/m², OAGB 28.3 ± 4.6 kg/m², RYGB 29.8 ± 4.9 kg/m²; *p* = 0.0005). Total weight loss (TWL) was also significantly higher in surgical groups (OMM 7.9% ± 8.4, OAGB 25.8%±11.2, RYGB 20.1%±10.5; *p* = 0.003). TWL ≥ 20% was achieved in 68.1% of OAGB patients, 46.7% of RYGB patients, and 9.4% of OMM patients (*p* < 0.001). Resolution of type 2 diabetes and hypertension was more frequent following surgical conversion. Revisional surgery rates were low and similar between surgical groups.

**Conclusions:**

Conversion surgery following SG was associated with greater long-term weight loss and metabolic improvement compared with OMM therapy alone. Among surgical options, OAGB demonstrated the highest proportion of patients achieving clinically meaningful weight loss.

## Key Points

- Recurrent weight gain after sleeve gastrectomy is a common long-term challenge requiring additional treatment strategies.
- Conversion surgery (OAGB or RYGB) resulted in significantly greater long-term weight loss compared with OMM.
- OAGB achieved the highest proportion of patients reaching clinically meaningful weight loss (TWL ≥ 20%).
- Conversion surgery was associated with higher rates of improvement in obesity-related diseases.

## Introduction

Metabolic and bariatric surgery (MBS) remains the most effective therapeutic option for patients with severe obesity [1]. Sleeve gastrectomy (SG) is currently the most widely performed procedure worldwide, largely due to evidence demonstrating durable weight loss, substantial improvement or resolution of obesity-associated medical problems, and reduced mortality [2–4].

Despite its extensive adoption, accumulating evidence indicates that SG carries a notable risk of recurrent weight gain and/or suboptimal clinical response. Published series consistently report weight regain rates ranging from 20 to 35%, with an increasing proportion of patients ultimately requiring revisional surgery [5]. In parallel, the volume of conversion procedures—most commonly to Roux-en-Y gastric bypass (RYGB) or one-anastomosis gastric bypass (OAGB)—has risen steadily in recent years [6–8]. These revisional operations are generally more technically demanding, associated with longer operative times, and carry higher perioperative risk compared with primary procedures [9]. In addition to managing recurrent weight gain, conversions are also performed for refractory gastroesophageal reflux disease (GERD), which remains a well-recognized late complication of SG [10].

In recent years, obesity management medications (OMMs) have rapidly gained prominence as an adjunct or alternative to MBS. The introduction of newer GLP-1 receptor agonists has transformed medical obesity care, offering superior efficacy compared with earlier agents while maintaining a favorable safety profile [11]. Their accessibility, scalability, and strong metabolic effects have contributed to widespread clinical adoption, including among patients after MBS [12, 13]. Current expert consensus increasingly supports a stepped-care approach in which patients experiencing recurrent weight gain after MBS initiate OMM therapy before considering conversion surgery [14]. This strategy aims to optimize weight control, potentially delay or obviate the need for conversion surgery, and reduce overall procedural risk. There is limited data regarding the long-term outcomes of OMM therapy after SG compared with conversion surgery following SG. This study aims to evaluate and compare the long-term results of OMM treatment versus conversion surgery (OAGB and RYGB) after SG.

## Methods

This study is a retrospective analysis of a prospectively maintained patient registry from a tertiary university hospital. All patients with recurrent weight gain after SG undergoing conversion surgery and/or under OMM treatment were included in the analysis (January 2014-December 2024). Exclusion criteria included age < 18 years, pregnancy, conversion surgery performed for indications other than recurrent weight gain, and loss to follow-up. Recurrent weight gain was defined as gaining > 30% of the nadir weight loss [15].

### Treatment Allocation

This was a retrospective study reflecting real-world clinical practice. Patients were referred either to the MBS clinic or to the endocrinology clinic for management of recurrent weight gain after SG. In our center, OMM management is conducted within the endocrinology clinic, which is an integral part of the obesity care center. Treatment decisions were not protocol-driven and were largely influenced by patient preference, with additional input from physician clinical judgment. In some cases, patients were subsequently referred between services based on treatment response. Patients in the OMM group were managed primarily in the endocrinology clinic.

## Patients’ Baseline Characteristics

Data captured for this study included patients’ baseline characteristics – type of conversion surgery, time from SG to conversion surgery, type of OMM, time from SG to OMM initiation, age, gender, body mass index (BMI) before SG, BMI before intervention, presence of obesity associated medical problems – type 2 diabetes (T2D), hypertension (HTN), Obstructive Sleep Apnea (OSA), and GERD.

Surgical considerations and technique – Indication for surgery comply with MBS indications according to local Ministry of Health guidelines (a body mass index [BMI] ≥ 40 kg/m², and BMI ≥ 35 kg/m² with one or more obesity related disease). All patients underwent a multi-disciplinary work-up and following that, the choice of conversion surgery was discussed, and a surgeon-patient decision was undertaken. RYGB was indicated in cases of Barrett’s esophagus, esophagitis grade C-D, large hiatal hernia, and in cases where reflux symptoms were significant. All other cases were referred to either RYGB or OAGB according to the surgeon-patient decision.

## OAGB Technique

The sleeved stomach was mobilized and divided using a linear stapler, with additional trimming performed when required, calibrated over a 36–40 Fr bougie (according to surgeon preference). The stomach was transected below the crow’s foot, creating a long, narrow gastric pouch, followed by a linear-stapled gastrojejunostomy constructed 150–200 cm distal to the ligament of Treitz.

## RYGB Technique

The sleeved stomach was mobilized and divided using a linear stapler, with additional trimming performed when required, calibrated over a 36–40 Fr bougie (according to surgeon preference). The stomach was transected above the incisura angularis, and a short gastric pouch was created along a 36–40 Fr bougie, an alimentary limb of 100 cm and a biliopancreatic limb (BPL) length of 100–150 cm were constructed using linear staplers. The mesenteric defects were closed routinely using non-absorbable sutures.

A blue dye leak test was performed routinely in all cases.

## Perioperative Outcomes

We captured data regarding the perioperative outcomes – type of conversion surgery, length of operative procedure, length of BPL, postoperative complications, grade of complications according to Clavien-Dindo classification [16], and reoperations.

### OMM Characteristics

Data capture included type of OMM, side effects, rate of OMM discontinuation, and rate of severe adverse events.

## Long Term Outcomes

Data captured regarding long-term outcomes included follow-up time, loss to follow-up, BMI at last follow-up, total weight loss (TWL). Total weight loss was calculated as: $\:(preoperative\:weight-last\:follow-up\:weight)/\left(preoperative\:weight\right)*100$.

Patients with TWL more or equal to 20% were considered satisfactory in terms of weight loss and the data was analyzed and compared accordingly [15]. In addition, data regarding resolution of T2D and HTN was withdrawn. We also included data regarding the need for additional interventions including surgical conversions/revisions and OMM use during the follow-up in all groups.

### Statistical Analysis

Continuous variables are presented as the mean ± standard deviation (SD). A normal distribution was assumed based on the central limit theorem. For continuous variables, differences between groups were assessed using the Brown-Forsythe and Welch ANOVA tests. To account for multiple comparisons, the two-stage Benjamini, Krieger, and Yekutieli False Discovery Rate (FDR) correction was applied. Categorical variables were compared using the chi-square test. A p-value < 0.05 was considered statistically significant. All statistical analyses were performed using GraphPad Prism (version 11 for macOS).

## Results

**Fig. 1:**
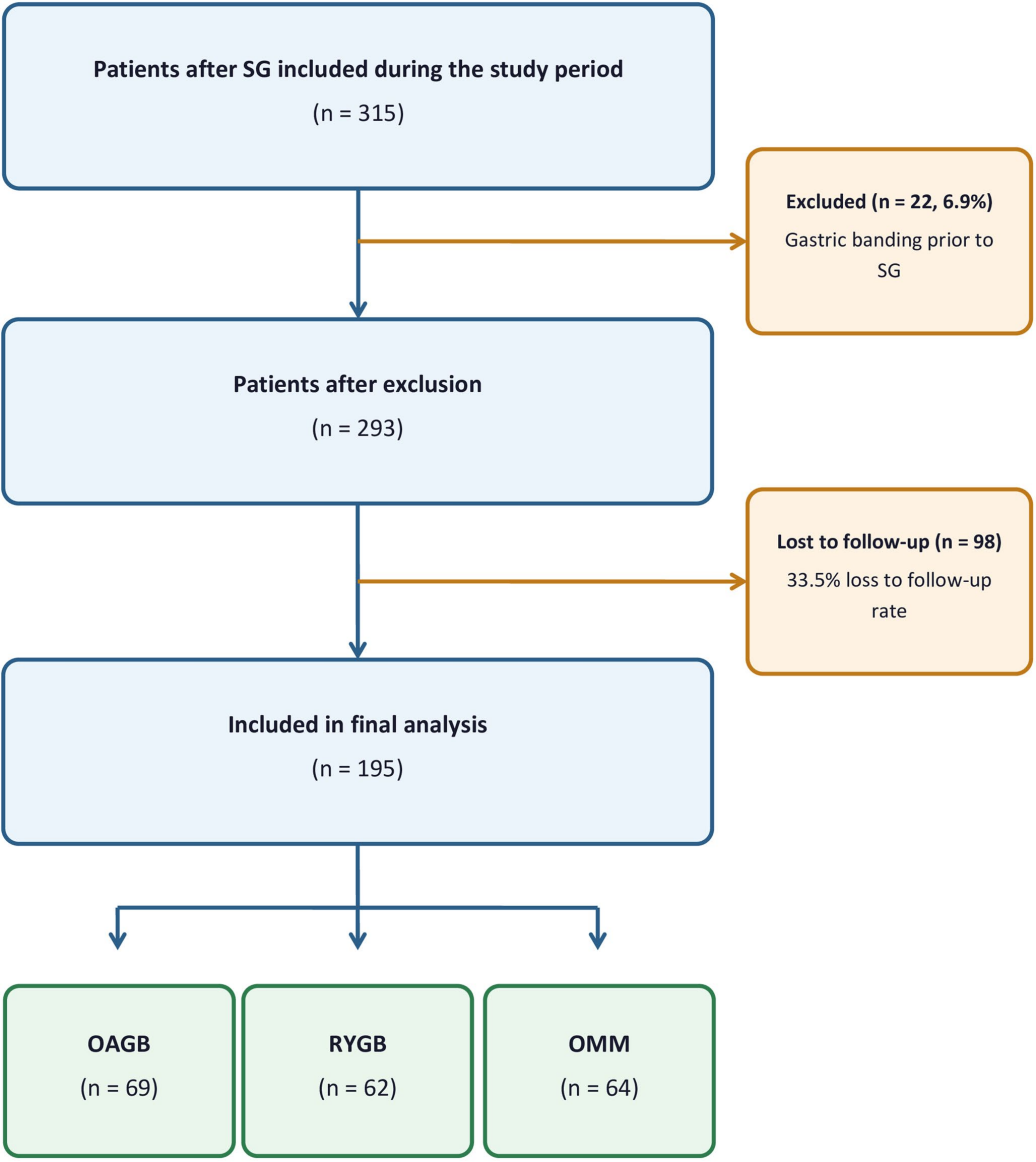
Flowchart of Patient Selection and Study Cohort. SG, sleeve gastrectomy; OAGB, one-anastomosis gastric bypass; RYGB,Roux-en-Y gastric bypass; OMM, obesity management medications

During the study period, 315 patients were initially included in the study. A flowchart illustrating patient selection, exclusions, and the final study cohort is presented in Fig. 1- Twenty-two patients (6.9%) underwent gastric banding prior to SG and were excluded, 98 patients were not available to follow-up (33.5% loss to follow-up rate), and eventually 195 patients were included in the final analysis. The study cohort included patients undergoing OAGB (*n* = 69), RYGB (*n* = 62), and OMM (*n* = 64).

Baseline characteristics of patients are shown in Table 1 – The mean age was significantly higher in the OMM group (OMM 55.9 ± 9.8, OAGB 48.8 ± 11.2, RYGB 53.9 ± 13.1, *p* = 0.0028). There were no gender differences between groups. The mean BMI before SG was insignificantly different between groups. The BMI prior to intervention was - OMM 36.2 ± 4.8 kg/m^2^, OAGB 38.2 ± 6.1 kg/m^2^, RYGB 37.3 ± 5.5 kg/m^2^, *p* = 0.11. There was no significant difference between groups in terms of obesity related medical problems except GERD which was significantly higher in patients undergoing RYGB (62.9% in RYGB vs. 42.1% in OAGB, and 31.3% in OMM; *p* = 0.001) and HTN was significantly higher in the OMM group (54.7% vs. 33.3% OAGB and 37.1% RYGB, *p* = 0.035).

**Table 1:** Baseline characteristics of patients undergoing conversion surgery or receiving OMMs after SG

Age (years)	OAGB (*n* = 69)	RYGB (*n* = 62)	OMM (*n* = 64)	*P* value
	48.8 ± 11.4	53.9 ± 13.1	55.9 ± 9.8	0.003
Males (n, %)	14 (20.3%)	20 (32.3%)	15 (23.4%)	0.27
BMI before SG (kg/m^2^)	48.6 ± 7.5	45.4 ± 6.9	47 ± 6.2	0.149
BMI at date of conversion surgery/intervention (kg/m²)	38.2 ± 6.1	37.3 ± 5.5	36.2 ± 4.8	0.11
T2D (n, %)	15 (21.7%)	14 (22.6%)	24 (37.5%)	0.08
HTN (n, %)	23 (33.3%)	23 (37.1%)	35 (54.7%)	0.035
OSA (n,%)	14 (20.3%)	7 (11.3%)	14 (21.9%)	0.25
GERD (n, %)	29 (42.1%)	39 (62.9%)	20 (31.3%)	0.001

*OMM* Obesity Management Medications, *SG* Sleeve Gastrectomy, *OAGB* One Anastomosis Gastric Bypass, *RYGB* Roux-en-Y Gastric Bypass, *BMI* Body Mass Index, *T2D* Type 2 diabetes, *HTN* Hypertension, *OSA* Obstructive Sleep Apnea, *GERD* Gastroesophageal Reflux Disease

Values are presented as mean ± standard deviation or percentage, as appropriate

*GERD* Gastroesophageal reflux disease, *SG* Sleeve Gastrectomy, *OAGB* One anastomosis gastric bypass, *RYGB* Roux-en-Y gastric bypass, *BMI* Body mass index, *T2D* Type 2 diabetes, *HTN* Hypertension, *HL* Hyperlipidemia, *OSA* Obstructive sleep apnea, *NAFLD* Non-alcoholic fatty liver disease, *PPI* Proton pump inhibitor

The mean time from SG to conversion surgery was 76.8 ± 9.77 months. Follow-up differed between groups, with shorter follow-up observed in the OMM group (OMM 61.3 ± 14.8 months vs. OAGB 83.7 ± 19.2 and RYGB 85.1 ± 17.6 months, *p* < 0.001).

The time of operative procedure in OAGB was 116 ± 52.5 min and 149 ± 58 min in RYGB with a statistically significant difference (*p* = 0.001). The mean BPL length was 194.3 ± 36.3 cm in OAGB and 121.7 ± 49 cm in RYGB. There were 2 major complications in the OAGB group and 2 in the RYGB group without a significant difference. Two patients in the RYGB required reoperation due to bleeding and two patients in the OAGB underwent laparoscopic drainage and lavage due to anastomotic leakage.

Patients in the OMM group were treated with GLP-1 receptor agonists including Semaglutide (*n* = 58) and Liraglutide (*n* = 8). During the treatment course, 11 patients received more than one obesity management medication. Side effects were reported in 18 patients (28.1%) and included: nausea (*n* = 12), constipation (*n* = 4), and vomiting (*n* = 2). While most side effects were mild and transient, five patients (8%) discontinued OMMs due to intolerance. No severe adverse events were recorded in the OMM group.

**Fig. 2:**
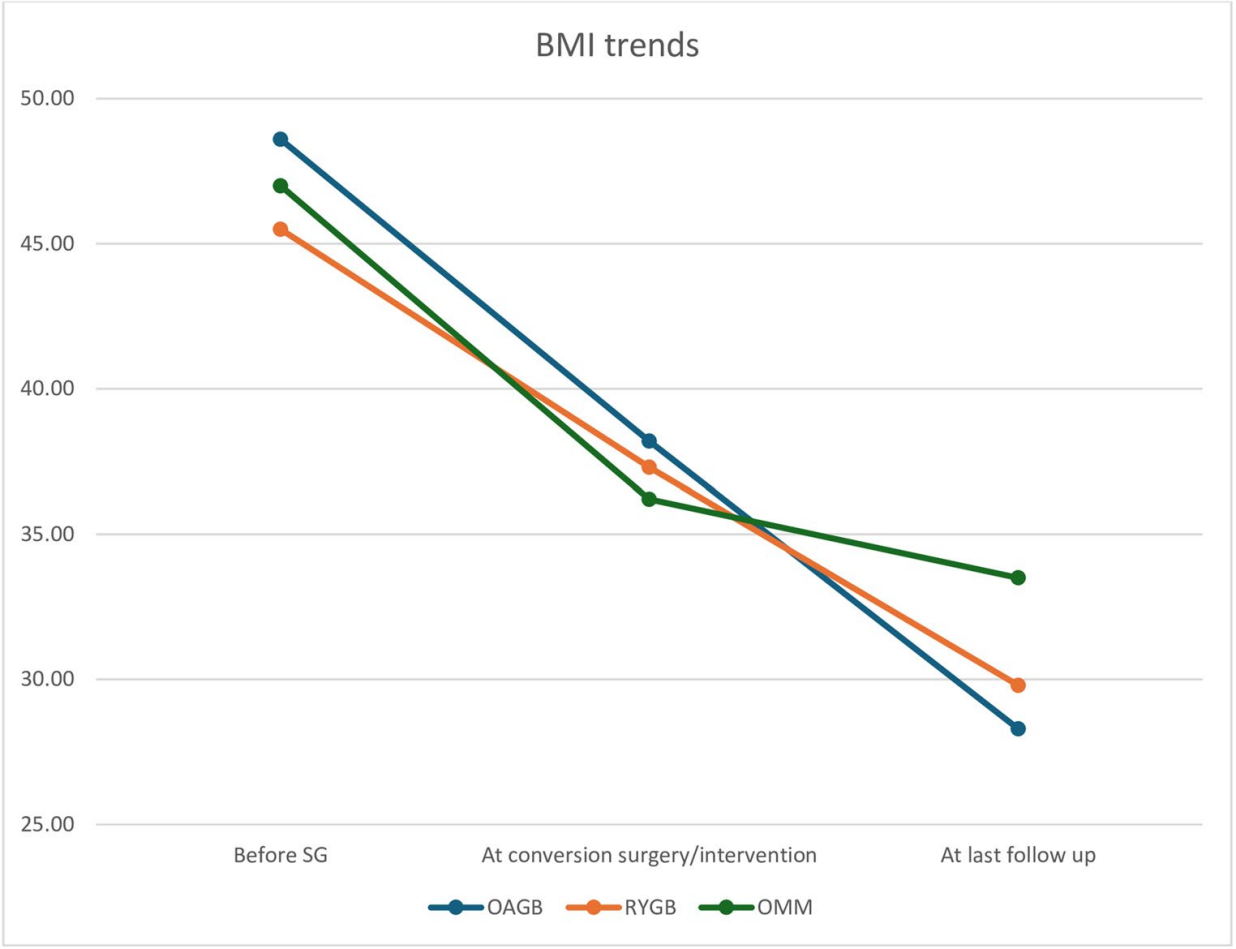
BMI trends of patients undergoing conversion surgery or receiving OMM after SG. BMI-Body Mass Index, OMM-Obesity Management, SG-Sleeve Gastrectomy, OAGB-One Anastomosis Gastric Bypass, RYGB-Roux-en-Y Gastric Byapass

The mean follow-up of patients was 76.8 months (6.4 years). The outcomes at follow-up are shown in Table 2 – There was a statistically significant difference in BMI at last follow-up between groups (OMM 33.3 ± 5.1, OAGB 28.3 ± 4.6, RYGB 29.8 ± 4.9; *p* = 0.0005) and TWL (OMM 7.9%±8.4, OAGB 25.8%±11.2, RYGB 20.1%±10.5%; *p* = 0.003). The rate of patients with TWL > 20% was significantly higher in the OAGB group (68.1%) versus the OMM (9.4%) and the RYGB group (46.7%) (*p* < 0.001). The BMI trends are shown in Fig. 2. Resolution rates of T2D and HTN were significantly higher in the OAGB and the RYGB group compared to the OMM group. During follow-up, the rate of revisional surgery was 5.8% in the OAGB group (marginal ulcer perforation – *n* = 2, anastomotic stricture – *n* = 1, insufficient weight loss – *n* = 1) and 6.4% in the RYGB group (Internal hernia *n* = 2, and fistula-related complications - *n* = 2) with no statistically significant difference between groups. During follow-up, a subset of patients in both surgical groups who were noted to initiate OMM treatment – 8 patients in the RYGB group (12.9%) and 6 patients in the OAGB group (8.7%).

**Table 2:** Outcomes of patients undergoing conversion surgery or receiving OMM after SG

Follow-up time (months) mean ± SD	OAGB (*n* = 69)	RYGB (*n* = 62)	OMM (*n* = 64)	*P* value
	83.7 ± 19.2	85.1 ± 17.6	61.3 ± 14.8	< 0.001
BMI at last follow-up (kg/m²)	28.3 ± 4.6	29.8 ± 4.9	33.5 ± 5.1	0.001
TWL (mean ± SD)	25.8 ± 11.2	20.1 ± 10.5	7.9 ± 8.4	< 0.001
TWL > 20% (n, %)	47 (68.1%)	29 (46.7%)	6 (9.4%)	< 0.001
T2D resolution (n, %) *	8/15	8/14	5/24	0.032
HTN resolution (n, %) *	13/23	9/23	8/35	0.037

*OMM* Obesity Management Medications, *SG* Sleeve Gastrectomy, *OAGB* One Anastomosis Gastric Bypass, *RYGB* Roux-en-Y Gastric Bypass, *BMI* Body Mass Index, *TWL* Total Weight Loss, *T2D* Type 2 Diabetes, *HTN* Hypertension

*The ratio was calculated from patients with preoperative diseases

## Discussion

In this study, we evaluated long-term outcomes of OMM compared with conversion MBS after SG in patients experiencing recurrent weight gain. Our findings demonstrate that conversion surgery resulted in significantly greater long-term weight loss compared with OMM therapy. At a mean follow-up of 6.4 years, BMI at last follow-up was 33.3 ± 5.1 kg/m² in the OMM group compared with 28.3 ± 4.6 kg/m² in the OAGB group and 29.8 ± 4.9 kg/m² in the RYGB group. TWL was 7.9%±8.4 with OMM treatment compared with 25.8%±11.2 after OAGB and 20.1%±10.5 after RYGB. The proportion of patients achieving clinically meaningful weight loss (TWL ≥ 20%) was 9.4% in the OMM group, 68.1% after OAGB, and 46.7% after RYGB. In addition, resolution of obesity related diseases such as T2D and HTN was more frequently observed following conversion surgery. These findings suggest that while OMM therapy may provide modest weight control, conversion MBS particularly OAGB—achieves more substantial and durable weight loss after SG.

Recurrent weight gain after SG represents a well recognized long term challenge. Published series report that approximately 15–35% of patients experience significant weight regain several years after SG [5, 17]. The mechanisms underlying this phenomenon are multifactorial and include behavioral factors, hormonal and metabolic adaptations, dilation of the gastric sleeve, and insufficient long-term lifestyle adherence [18]. Traditionally, management strategies for recurrent weight gain have relied on conversion MBS, most commonly conversion to RYGB or OAGB, both of which have demonstrated favorable weight loss and metabolic outcomes [19, 20]. However, conversion surgery is technically more complex and carries a higher perioperative risk compared with primary procedures [21]. In recent years, OMMs have emerged as an additional therapeutic option for patients experiencing recurrent weight gain after MBS. The introduction of highly effective agents, particularly GLP-1 receptor agonists such as semaglutide, has significantly expanded medical treatment possibilities and led to increasing use of pharmacotherapy as an adjunct or alternative to conversion surgery. As a result, contemporary management of recurrent weight gain after SG increasingly involves a multimodal approach incorporating pharmacological strategies [22].

Several studies have demonstrated that OMMs can induce modest but clinically meaningful weight loss in patients after MBS and may help stabilize recurrent weight gain in selected cases; In a systematic review and meta-analysis on the role of GLP-1 agonists in weight regain after bariatric by Mousavi et al. [23], the analysis showed greater weight loss in the GLP-1 group than placebo also for patients receiving < 6 months treatment. Additionally, Nie et al. reported that at least 3 months of treatment, the pooled %TWL was 9.24% for liraglutide, 11.38% for semaglutide, and 15.50% for tirzepatide [24]. Medical therapy also offers several advantages, including avoidance of operative risk, lower immediate morbidity, and easier implementation in routine clinical practice [25]. In our cohort, patients treated with OMMs were older and had a higher prevalence of hypertension, suggesting that this group may have represented less optimal surgical candidates, reflecting real-world selection bias in treatment allocation.

However, important limitations remain to OMMs. Besides inferior weight loss outcomes, treatment is often associated with high rates of gastrointestinal side effects, and long term adherence may be limited; in a prospective cohort by Almohaileb et al. [26], the most commonly reported reasons for treatment discontinuation included side effects (36%), logistical challenges such as geographic relocation, inflexible work schedules, or limited transportation access (24%), medication cost (23%), supply shortages (11%), and dissatisfaction with weight loss response (7%). In addition, the high cost of these medications and the need for prolonged or lifelong treatment raise important economic considerations for both patients and healthcare systems [27]. Given the expanding role of OMMs in the management of recurrent weight gain after MBS, careful patient selection and coordinated long-term follow-up are essential. Costs are also an important consideration when comparing these approaches. OMMs, particularly GLP-1 receptor agonists, often require long-term use and can be associated with significant ongoing costs, which may affect accessibility and adherence. In contrast, although conversion surgery involves higher upfront costs, its more durable effect may make it more cost-effective over time. While we did not formally assess this in the current study, it is an important factor to consider in clinical decision-making. In this context, management by a dedicated multidisciplinary team is particularly important to ensure appropriate treatment selection, optimize adherence, and facilitate timely escalation to surgical intervention when medical therapy is insufficient.

Our findings are consistent with previous reports demonstrating favorable outcomes of conversion surgery following sleeve gastrectomy. Several studies have shown that conversion to OAGB or RYGB after SG is associated with significant additional weight loss and improvement in obesity-related diseases. In a systematic review and meta-analysis of comparative studies by Vitiello et al. [28], OAGB showed superior results when compared to RYGB in terms of weight loss and GERD remission trends were better in patients after RYGB. Felsenreich et al. [29] reported total weight loss of patients converted to RYGB and OAGB was 41.5% and 44.8%, respectively, at nadir. Interestingly, 30% of patients after RYGB still had symptomatic GERD, while 53.8% of patients after OAGB had symptomatic GERD. In another meta-analysis comparing OAGB to RYGB after SG, weight loss outcomes were similar and GERD resolution was higher in patients after RYGB [30]. In a study reporting outcomes up to 14 years after conversion of SG to RYGB, total weight loss was approximately 15% at last follow-up, with a clear trend toward weight regain observed after 3–4 years [31]. Reported total weight loss after conversion procedures typically ranges between 15% and 40%, depending on the type of conversion procedure and duration of follow-up. Conversion to OAGB has been reported in several studies to provide slightly higher weight loss outcomes following SG, likely due to its stronger hypoabsorptive component and longer biliopancreatic limb [32, 33]. In contrast, RYGB is generally considered the preferred revisional procedure in patients with significant gastroesophageal reflux disease after SG, as it offers effective reflux control through diversion of bile and acid away from the esophagus [29, 34]. Therefore, the choice between OAGB and RYGB should be individualized, taking into account the primary indication for revision, particularly the relative importance of weight loss versus reflux control, as well as patient characteristics and surgeon experience. In the present study, patients undergoing OAGB achieved a mean TWL of 25.8%, while those undergoing RYGB achieved a TWL of 20.1%, findings that fall within the range reported in previous series. In contrast, weight loss achieved with OMM therapy in our cohort was more modest, with a mean TWL of 7.9%. While pharmacotherapy may represent a reasonable initial strategy in selected patients, our results suggest that conversion surgery remains the most effective option for achieving substantial and durable weight loss in patients with significant recurrent weight gain after SG.

The findings of the present study have several important clinical implications for the management of patients experiencing recurrent weight gain after SG. While OMMs may provide a non-invasive option and may help address recurrent weight gain in selected patients, our results suggest that their overall weight loss effect remains limited compared with surgical conversion. Conversion procedures, particularly OAGB and RYGB, provide significantly greater and more durable weight loss and higher rates of improvement in obesity-related diseases. Therefore, the choice between pharmacological therapy and conversion surgery should be individualized based on the severity of weight regain, presence of obesity related diseases, patient preference, and surgical risk. In clinical practice, a stepwise approach may be reasonable, where OMMs are initially considered in patients with mild or moderate weight regain, while conversion surgery may be more appropriate for patients with significant weight regain or inadequate response to medical therapy. However, this approach was not directly evaluated in our study and should be interpreted with caution pending further prospective validation. The observed cross-over rate of 8.7–12.9% is clinically relevant. In our cohort, this was not a planned adjunctive strategy but rather reflects real-world practice. We believe this trend is driven by the increasing availability and widespread use of OMMs in public healthcare settings. As such, treatment is often sequential, with patients transitioning between modalities due to suboptimal response, rather than as part of a predefined combined approach. While this may introduce some degree of confounding, these cases were retained to better represent clinical reality.

This study has several limitations that should be acknowledged. First, the study was retrospective in nature and conducted at a single tertiary referral center, which may introduce selection bias. Second, although the registry was prospectively maintained, treatment allocation to OMM therapy or conversion surgery was not randomized and was influenced by clinical decision-making and patient preference. Third, the follow-up rate was relatively low (~ 70%) and follow-up duration differed between groups, with shorter follow-up observed in the OMM group, which may influence the interpretation of long-term outcomes. In addition, the study included a relatively heterogeneous population in terms of type and duration of pharmacological treatment. In addition, detailed data on treatment duration, adherence, and continuity were not consistently available, and outcomes may therefore reflect periods of treatment interruption. This should be considered when comparing these results with conversion surgery. Nevertheless, the study provides valuable long-term comparative data on the outcomes of OMMs versus conversion surgery after SG in patients with recurrent weight gain. Despite the relatively high loss to follow-up, the remaining cohort still represents one of the larger series evaluating long-term outcomes in this setting.

## Conclusions

In conclusion, recurrent weight gain after SG remains a significant long-term challenge requiring effective management strategies. In the present study, conversion MBS demonstrated greater and more durable weight loss compared with OMM alone, with OAGB achieving the highest proportion of clinically meaningful weight loss. While pharmacological therapy may represent a reasonable initial approach in selected patients, conversion surgery remains the most effective option for patients with significant recurrent weight gain after SG.

### Compliance with ethical requirements

## Data Availability

No datasets were generated or analysed during the current study.
